# Beyond Return‐To‐Sport: Mapping ACL Injury History in Currently Active Female Football Players From Youth to Senior Elite Level

**DOI:** 10.1155/tsm2/4174828

**Published:** 2026-04-28

**Authors:** Mette K. Zebis, Mikkel B. Clausen, Connie Linnebjerg, Mette Hansen, Jesper Bencke, Lars L. Andersen, Per Hölmich, Kristian Thorborg

**Affiliations:** ^1^ Department of Midwifery, Physiotherapy, Occupational Therapy and Psychomotor Therapy, Faculty of Health, University College Copenhagen, Copenhagen, Denmark, ucc.dk; ^2^ Institute of Sports Medicine Copenhagen, Copenhagen University Hospital Bispebjerg and Frederiksberg, Copenhagen, Denmark; ^3^ Department of Public Health, Aarhus University, Aarhus, Denmark, au.dk; ^4^ Human Movement Analysis Laboratory, Department of Orthopedic Surgery, Copenhagen University Hospital, Amager-Hvidovre, Copenhagen, Denmark, gentoftehospital.dk; ^5^ National Research Centre for the Working Environment, Copenhagen, Denmark, arbejdsmiljoforskning.dk; ^6^ Department of Health Science and Technology, Aalborg University, Aalborg, Denmark, aau.dk; ^7^ Sports Orthopedic Research Center–Copenhagen (SORC-C), Department of Orthopaedic Surgery, Copenhagen University Hospital, Amager-Hvidovre, Copenhagen, Denmark, gentoftehospital.dk

**Keywords:** ACL injury, knee pain, playing level, soccer, women

## Abstract

**Purpose:**

This study determines the prevalence of ACL injury history among currently active female football players across age groups and playing levels and examines the association between ACL injury history and activity‐related knee pain.

**Methods:**

A total of 1026 active Danish female youth‐ and senior‐league football players were invited to an online questionnaire (response rate: 751 [73%]) on (1) ACL injury history and (2) present knee pain during physical activity. Prevalence was calculated separately according to age (youth vs senior) and playing level (non‐elite vs elite). Logistic regression analyses investigated factors associated with the prevalence of ACL injury history and activity‐related knee pain, respectively.

**Results:**

The prevalence of ACL injury history was 5.0% (95% confidence interval [CI] 3.3%–7.4%) in youth football and 14.8% (95% CI 10.6%–19.8%) in senior football. The prevalence of activity‐related knee pain was comparable between youth (24.6%, 95% CI 20.8%–28.7%) and senior football players (26.0%, 95% CI 20.7%–31.9%). Comparable prevalence of ACL injury history and knee pain was reported at elite and non‐elite level. ACL injury history was strongly associated with activity‐related knee pain (OR = 5.4–8.7, *p* < 0.0001).

**Conclusion:**

Playing with a previous ACL injury is common in active female football players, particularly at senior levels (nearly 1 in 6 elite players). The strong association between ACL injury history and activity‐related knee pain underscores the long‐term negative consequences. This study highlights the need for secondary and tertiary prevention strategies in female football.

## 1. Introduction

Female football is associated with one of the highest rates of anterior cruciate ligament (ACL) injuries [1, 2] and represents the highest injury burden [3, 4]. An ACL injury represents a life‐changing event with immediate effects on sports participation, physical activity, and quality of life [5, 6]. Furthermore, higher risk of knee pain, impaired knee function, and early development of osteoarthritis are also severe long‐term consequences of an ACL injury sustained during youth or senior football [7, 8]. Furthermore, many ACL‐injured athletes do not regain their preinjury level: Only ∼50% of football players return to football [9, 10], and female players have a 5‐fold higher rate of new ACL injuries compared to male players [11].

Within the framework of injury prevention, the first step is to establish the extent of the problem [12, 13]. When reporting ACL injury risk in sports, it is often expressed as incidence (e.g., 0.08/1000 playing hours [14]). A previous review summarized the annual incidence rate to be between 0.5% and 6.0% of female football players who incur an ACL injury during a match season [15], and recent reports show that elite teams in Europe can expect 0.7 ACL injuries per season [3]. However, as an ACL injury may be considered a permanent injury due to long‐lasting and recurrent (chronic) symptoms, another important measure is prevalence, which is the proportion of a population that has the characteristic (i.e., ACL injury history).

The point prevalence is especially relevant to report among active football players, because, at a given time point (e.g., at the start of the season), it uncovers the extent of players still playing who have suffered this specific injury [16], and where both primary, secondary, and tertiary injury prevention initiatives should take place. The point prevalence can be extracted from prospective or interventional studies that report the number of players with an ACL injury history at baseline, and has previously been reported to vary between 0.9% [17] and 18.4% [18] at the start of the season in a given population of female football players. However, it is not clarified whether the odds of having an ACL injury history are equal among youth vs. senior female football players and among non‐elite vs. elite players. Furthermore, there is a lack of knowledge regarding the prevalence of activity‐related knee pain and how pain is associated with previous ACL injury in a population of active female football players.

Thus, the aim of the present cross‐sectional survey was to report the point prevalence of ACL injury history at preseason (i.e., a comparable time point across teams for assessing players before match season) among active playing female football players and to estimate the odds of having an ACL injury history in relation to age (youth vs. senior players) and playing level (non‐elite vs. elite players). Further aims were to report the prevalence of players (non‐elite vs. elite players; youth vs. senior players) who experience activity‐related knee pain and to investigate whether activity‐related knee pain was associated with ACL injury history.

## 2. Materials and Methods

### 2.1. Study Design, Subjects, and Data Collection

This study is a cross‐sectional questionnaire survey among female football players. The study conforms to all STROBE guidelines and reports the required information accordingly (Supporting Information 1). The survey reports the prevalence of ACL injury as well as knee pain and discomfort during different physical activities. Data were collected at two occasions, i.e., Spring 2012 and Spring 2019, in Denmark. In the 2012 survey, an online questionnaire was sent out by mail to 498 adolescent female football players, for whom the injury rates had been reported previously [19, 20]. The data collected in the 2012 survey provided insights into pursuing the same type of information to a larger extent—i.e., across age groups and playing level. Thus, the initial 2012 survey was expanded with the 2019 survey in the form of an online questionnaire sent out by mail to 528 adolescent and adult female football players.

In the 2012 survey, players were considered eligible for inclusion if they were enrolled in a female football team in the age group U18 (15–18 years), resided in Jutland (a specific region of Denmark), and participated in any Danish Football Association (DBU) series during the 2012 season. In the 2019 survey, players were considered eligible for inclusion if they were enrolled in a female football team in the best national youth league (U18DM) or the two highest national senior leagues in Denmark (1. Division and Gjensidige League). All players (and their parents if the players were below 18 years old) were informed about the study, and written consents, signed by the player or the player’s legal guardian, were collected. The 2012 survey was approved by the National Committee on Health Research Ethics in Denmark (H‐2‐2010‐091). The 2019 survey was, by the National Committee on Health Research Ethics in Denmark, not considered to require full ethical evaluation (H‐19010648). Both surveys were conducted in accordance with the Helsinki Declaration (World Medical Association, 2012).

### 2.2. Questionnaire

From the questionnaire, we extracted demographic data, football experience, playing level, knee injury history, and knee pain or discomfort during four different activities (i.e., football training, football match, leisure time, and work/education).

#### 2.2.1. Non‐Elite Versus Elite and Youth Versus Senior

Based on self‐reported team participation in the national leagues, four categories were identified: (1) senior elite ≥ 18 years, (2) senior non‐elite ≥ 18 years, (3) youth elite < 18 years, and (4) youth non‐elite < 18 years. The elite level was defined as participation in the best national league for < 18 years (U18DM) and ≥ 18 years (Gjensidige league), respectively. For the statistical analyses, age groups were defined as senior (i.e., non‐elite and elite ≥ 18 years) and youth (i.e., non‐elite and elite < 18 years). Playing level was defined as elite level, including elite ≥ 18 years and elite < 18 years, and non‐elite level, including non‐elite ≥ 18 years and non‐elite < 18 years.

### 2.3. ACL Injury

To identify players with an ACL injury history, each player replied to the following question: *“Have you ever had a knee injury related to playing football?”* If the player answered *“yes,”* the next question was: *“Specify which type of knee injury”*—with the corresponding options *(1) ACL*—*right knee, (2) ACL*—*left knee, (3) Meniscus*—*right knee, (4) Meniscus*—*left knee, and (5) Others (text field).* It was possible for the player to choose more than one option. If the player chose Options 1 and/or 2, the player also replied to the number of ACL injuries in the right and left knee, respectively, and the time since the most recent ACL injury. Based on the replies to the above question, two groups were identified: (1) players with ACL injury history (ACL) and (2) players with no ACL injury history (No ACL).

### 2.4. Knee Pain and Discomfort

The players answered yes/no to the following question: *“Do you experience pain or discomfort in your knee during the following activities (1) football match playing, (2) football training, (3) work or education, and/or (4) leisure time.”* It was possible for the player to choose more than one option. Based on the replies to the above questions, two groups were identified: (1) players who reported knee pain during one or more activities (Knee pain) and (2) players who did not report knee pain during any of the mentioned physical activities (No knee pain).

### 2.5. Sample Size Consideration

Previously, the prevalence of female youth and senior football players with an ACL injury history has been reported to be 4% and 18.3% [18], respectively. Assuming this, the required sample size to detect a difference between youth and senior football in the present survey was 178 football players (89 youth players and 89 senior players), when using a two‐population proportion formula for cross‐sectional study design with the following assumptions: Two‐sided confidence level = 95%; power: 80%; ratio = 1:1.

### 2.6. Statistical Analyses

Data analyses were performed with SPSS (v 26; IBM Inc.). The data analyses consisted of descriptive statistics and regression modeling. The ACL injury and knee pain prevalence, respectively, were the number of individuals reporting the event divided by total number of respondents, presented as percentage and 95% confidence intervals (CIs). Binary logistic regression analysis was performed to determine the association between ACL injury prevalence proportion (outcome variable) and age level (youth vs senior), playing level (non‐elite vs elite), and BMI (continuous variable). A second analysis was performed to determine the association between knee pain or discomfort during four different activities (outcome), respectively, and age level (youth vs senior), playing level (non‐elite vs elite), BMI (continuous variable), and ACL injury (no vs yes).

In the analyses, the dependent variables were defined as (1) ACL status (0 = normal; 1 = injury) and (2) knee pain or discomfort (0 = no; 1 = yes). Odds ratios (ORs) and their respective 95% CIs were estimated. An *α* level of 0.05 was accepted as statistically significant.

### 2.7. Patient and Public Involvement

No patient (player) or public involvement took place in the design or planning of the study. No funders played a role in the design, conduct, or reporting of this study.

## 3. Results

### 3.1. Response Rate

**FIGURE 1:**
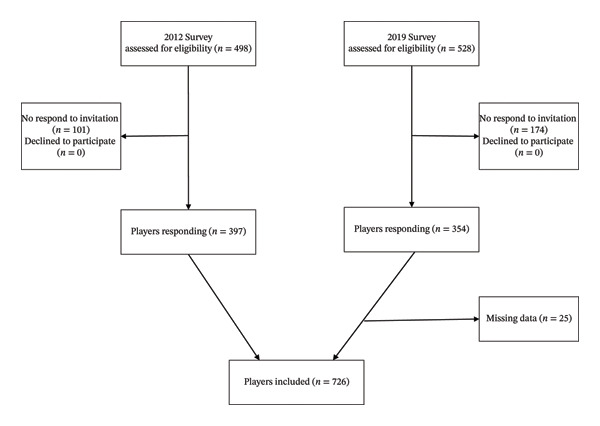
Flow of participants in the study.

In total, 751 players answered the online questionnaires (response rate 73%), representing 121 different teams. Eight players were included in both surveys. Of the 751 players, 25 players did not answer all questions included in this study and were excluded from the analyses, resulting in a final sample of 726 players (Figure 1). The 25 players with missing data had a history of three ACL injuries. Table 1 shows characteristics of the included players.

**TABLE 1:** Characteristics of female football players.

	**No ACL (*n* = 665)**	**ACL (*n* = 61)**	**No knee pain (*n* = 544)**	**Knee pain (*n* = 182)**

Demographics				
Age (y)	19 (±4)	21 (±5)	19 (±4)	19 (±4)
Height (cm)	168 (±6)	169 (±6)	168 (±6)	169 (±6)
Weight (kg)	60 (±7)	63 (±7)	60 (±7)	61 (±7)
Body mass index (kg^.^m^−2^)	21 (±2)	22 (±2)	21 (±2)	19 (±2)
Playing experience (y)	9 (±6)	12 (±7)	9 (±6)	9 (±6)

*Note:* Data presented as mean ±SD. No ACL: Players with no previous ACL injury history. ACL: Players with an ACL injury history. No knee pain: Players who report no knee pain during physical activity. Players who report knee pain during physical activity.

Abbreviation: ACL, anterior cruciate ligament.

### 3.2. Prevalence of Players With an ACL Injury History

Overall, 61 players (8.4%, 95% CI 6.5% to 10.7%) had a history of 64 ACL injuries (three non‐elite players had bilateral ACL injury, i.e., one senior player and two youth players). Time from injury was within two years for 59% of the ACL‐injured players, with five players being within six months postinjury at the time of completing the survey. Of the 61 players reporting an ACL injury history, 16 players had a combined ACL/meniscus injury history.

With respect to age and playing level, the prevalence of players with an ACL injury history is presented in Table 2.

**TABLE 2:** Prevalence of ACL injury history.

Overall	No ACL (*n*)	ACL (*n*)	Prevalence	95% CI
	665	61	8.4%	(6.5%–10.7%)
Youth	452	24	5.0%	(3.3%–7.4%)
Senior	213	37	14.8%	(10.6%–19.8%)
Non‐elite	473	37	7.3%	(5.2%–9.9%)
Elite	192	24	11.1%	(7.3%–16.1%)
Youth non‐elite < 18 years	324	14	4.1%	(2.2%–6.9%)
Youth elite < 18 years	128	10	7.2%	(3.5%–12.9%)
Senior non‐elite ≥ 18 years	149	23	13.4%	(8.7%–19.2%)
Senior elite ≥ 18 years	64	14	17.9%	(10.2%–28.3%)

Abbreviations: ACL, anterior cruciate ligament; CI, confidence interval.

Playing at the senior level (≥ 18 years) increased the odds of an ACL injury history relative to the youth level (OR = 2.8, 95% CI 1.6 to 4.9), while odds of an ACL injury history did not differ significantly between elite and non‐elite players, Table 3.

**TABLE 3:** Factors associated with the prevalence of ACL injury.

		**Previous ACL injury**		
		**OR**	**95% CI**	*p* **-value**

Factors:				
Age	Youth	1		
	Senior	2.8	1.6–4.9	< 0.001

Level (non‐elite vs elite)	Non‐elite	1		
	Elite	1.6	0.9–2.8	0.092

BMI	(per unit)	1.1	1.0–1.3	0.061

Abbreviations: ACL, anterior cruciate ligament; BMI, body mass index; CI, confidence interval; OR, odds ratio.

### 3.3. Prevalence of Players With Activity‐Related Knee Pain

**FIGURE 2:**
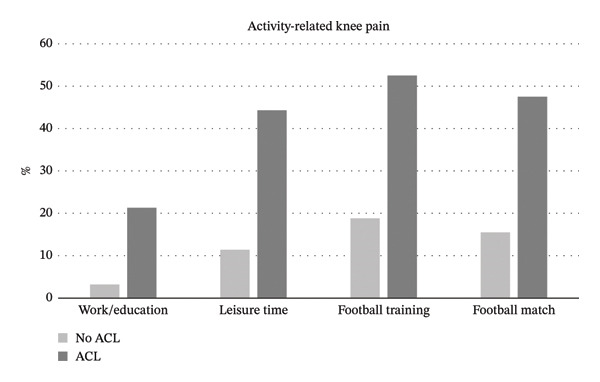
Activity‐related knee pain among 1) players who reported an ACL injury history (ACL, dark bars) and players with no previous ACL injury history (No ACL, light bars).

The overall prevalence of knee pain during physical activity was 25.1% (95% CI 22.0% to 28.4%) and was similar between youth (24.6%, 95% CI 20.8%–28.7%) and senior football players (26.0%, 95% CI 20.7%–31.9%), and between elite (21.8%, 95% CI 16.5%–27.9%) and non‐elite (26.5%, 95% CI 22.7%–30.5%) football players. The prevalence of players with knee pain during football matches, football training, work/education, and leisure time, respectively, is presented in Figure 2 and Table 4. An ACL injury history increased the odds of reporting knee pain regardless of the type of physical activity (OR = 5.4–8.7) (Table 5). The prevalence of activity‐related knee pain did not differ between youth and senior football players or between elite and non‐elite football players, see Table 4. With regard to specific categories of activity‐related pain, age (youth vs senior), level (non‐elite vs elite), and BMI (continuously variable) did not significantly influence the odds for reporting pain during training, matches, and work/education (Table 5). However, playing at non‐elite vs elite level increased the odds of knee pain during leisure time activity by 2.7‐fold (95% CI 1.5 to 4.8), Table 5.

**TABLE 4:** Prevalence of knee pain during physical activity with 95% confidence interval.

Overall (*n* = 726)	Work/education	Leisure time	Football training	Football match
	4.7% (3.3%–6.5%)	13.8% (11.4%–16.5%)	21.6% (18.7%–24.8%)	18.2% (15.4%–21.2%)
Youth (*n* = 476)	4.2% (2.6%–6.4%)	14.5% (11.5%–18.0%)	21.4% (17.8%–25.4%)	18.5% (15.1%–22.3%)
Senior (*n* = 250)	5.6% (3.1%–9.2%)	12.4% (8.6%–17.1%)	22.0% (17.0%–27.7%)	17.6% (13.1%–22.9%)
Non‐elite (*n* = 510)	5.1% (3.4%–7.4%)	16.3% (13.2%–19.8%)	22.8% (19.2%–26.6%)	19.0% (15.7%–22.7%)
Elite (*n* = 216)	3.7% (1.6%–7.2%)	7.9% (4.7%–12.3%)	19.0% (14.0%–24.9%)	16.2% (11.6%–21.8%)
Youth non‐elite < 18 years (*n* = 338)	5.0% (3.0%–7.9%)	17.5% (13.6%–21.9%)	22.8% (18.4%–27.6%)	18.6% (14.6%–23.2%)
Youth elite < 18 years (*n* = 138)	2.2% (0.5%–6.2%)	7.3% (3.5%–12.9%)	18.1% (12.1%–25.6%)	18.1% (12.1%–25.6%)
Senior non‐elite ≥ 18 years (*n* = 172)	5.2% (2.4%–9.7%)	14.0% (9.2%–20.1%)	22.7% (16.7%–29.7%)	19.8% (14.1%–26.5%)
Senior elite ≥ 18 years (*n* = 78)	6.4% (2.1%–14.3%)	9.0% (3.7%–17.6%)	20.5% (12.2%–31.2%)	12.8% (6.3%–22.3%)

**TABLE 5:** Factors associated with knee pain or discomfort during work/education, leisure time, training, and match.

		**Work/education**			**Leisure time**			**Training**			**Match**		
		**OR**	**95% CI**	**p** **-value**	**OR**	**95% CI**	**p** **-value**	**OR**	**95% CI**	**p** **-value**	**OR**	**95% CI**	**p** **-value**

Factors:													
Age	Youth	1			1			1			1		
	Senior	1.2	0.5–2.6	0.662	0.6	0.4–1.0	0.057	0.9	0.6–1.8	0.656	0.8	0.5–1.2	0.221

Level	Non‐elite	1			1			1			1		
	Elite	0.6	0.3–1.4	0.237	0.4	0.2–0.7	< 0.001	0.7	0.5–1.1	0.115	0.7	0.5–1.2	0.183

ACL injury	No	1			1			1			1		
	Yes	8.7	3.9–19.3	< 0.001	6.7	3.6–12.4	< 0.001	5.4	3.1–9.6	< 0.001	5.7	3.2–10.1	< 0.001

BMI	(per unit)	1.1	0.9–1.3	0.377	1.0	0.9–1.1	0.690	0.9	0.9–1.0	0.167	1.0	0.9–1.1	0.692

Abbreviations: ACL, anterior cruciate ligament; BMI, body mass index; CI, confidence interval; OR, odds ratio.

## 4. Discussion

This is the first study to report the prevalence of an ACL injury history concurrent with the prevalence of knee pain during physical activities in a population of both active playing youth and senior female football players. The prevalence of players with an ACL injury history was highest at the senior level, whereas the prevalence of knee pain was similar between age groups and playing level. Thus, the odds of having an ACL injury history in the senior group were almost three times that observed in the youth group. ACL injury history was strongly associated with reporting knee pain during football as well as during work and leisure‐time activities.

### 4.1. Prevalence of ACL Injury History

Overall, 8.4% female football players had a history of 64 ACL injuries. Especially, the senior elite level demonstrated a high prevalence, i.e., ∼18% of the players in the best national football league had a history of ACL injury. In comparison, ∼8% of male elite football players have been reported to have an ACL injury history [21]. In a study among American female football players, the prevalence of elite senior players with an ACL injury history was comparable with our data and was found to be significantly higher compared with elite youth players (18.3% vs 4.0%; *p* < 0.001) [18]. In our survey, the prevalence of senior elite vs. youth elite female football players with an ACL injury history was not found to be significantly different (17.9%; 95% CI, 10.2%–28.3% vs. 7.2%; 95% CI, 3.5%–12.9%), though point estimates reveal a difference of 10.7% in our sample. The prevalence of players with an ACL injury history in youth non‐elite football was 4.1%. Previously, the prevalence of ACL injury among Swedish non‐elite youth female football players (aged 12–17 years) has been reported to be less than 0.9% of all players [17]. The lower prevalence reported by Waldén et al. [17] may partly be explained by the fact that the cohort includes 12‐ to 14‐year‐old players who are less represented in the ACL injury statistics compared to the age group 15–18 years [22].

In this survey, playing at senior level (≥ 18 years) was significantly associated with a higher prevalence of ACL injuries (Table 3). Since the ACL injury incidence is reported to be highest at youth level [22], the higher prevalence observed at senior level in the present study may partly be explained by an accumulation of former youth players who succeeded in returning to football after an ACL injury and who continue to play into the senior level.

### 4.2. Activity‐Related Knee Pain

The overall prevalence of players who reported knee pain in one or more of the four categories of physical activity was 25.1%. As far as we know, no other study has described the prevalence of knee pain based on similar questions, making a direct comparison with other studies difficult. Nonetheless, the three‐month prevalence of overall knee pain among Danish employees in the age group 18–25 years was 27.7% (Work Environment and Health in Denmark Study 2012–2018, unpublished data), which corresponds closely to the present finding. Furthermore, in a Danish cohort of 2200 adolescents (aged 15 to 19 years), 504 reported knee pain, corresponding to a prevalence of 22.9% [23]. Hence, it seems that knee pain is a common phenomenon in the general population with approximately one in four reporting this. An interesting finding in our study was that non‐elite players reported a higher prevalence of knee pain during leisure‐time activities than elite players, and this was the only activity category in which a between‐group difference was observed. One possible explanation could be that elite players—due to higher weekly football exposure—may engage in less physically demanding activities during their leisure time, thereby reducing their likelihood of experiencing knee discomfort.

Activity‐related knee pain was highly prevalent in players with an ACL injury history, i.e., one in two players reported knee pain during football training/match. In line, pain during physical activity was reported to be the most common symptom/problem in football players three years after ACL reconstruction [9]. Myklebust et al. reported in a 1‐ to 6‐year follow‐up among elite handball and football players that KOOS scores were significantly lower for ACL‐injured knees compared to knees of noninjured players [24]. Similarly, Ezzat et al. reported poorer KOOS values on all subscales in previously ACL‐injured athletes compared to age‐ and sports‐matched controls [25].

Our study further indicates that knee pain or discomfort during work/education is significantly more prevalent in the group of previously ACL‐injured players compared to their noninjured peers. The fact that these players are of a young age with a long working life ahead of them underlines the severity of an ACL injury and takes the problem beyond the criterion of successful return to sports.

### 4.3. Implications

The prevalence data underline that not only primary prevention, but also initiatives for secondary and tertiary prevention are highly needed in female football. The need for preventive initiatives is further stressed by the fact that the ACL injury rate has increased during the last two decades in female football [26], whereas the injury incidence has been reported to gradually decrease [27] or remain unchanged [28] in male elite football during the last 20 years. In this context, it is important to emphasize that the majority of studies investigating the efficacy of injury prevention interventions are performed in male football [29], and only low‐level evidence exists on how to reduce ACL injuries in female football [30].

### 4.4. Limitations

First, as the players were asked to self‐report previous traumatic knee injuries related to football, the study design may introduce injury recall bias [31]. However, the severity of an ACL injury with subsequent medical attention and surgery minimizes the risk of recall bias. Although information on the chosen treatment method (surgical vs. conservative) was not collected in the survey, standard clinical practice in Denmark for female athletes aiming to return to pivoting sports is ACL reconstruction.

Second, the present survey was cross‐sectional and did not include individual‐level data to elucidate whether the high prevalence of ACL injury at the senior level represents former youth players who succeeded in returning to football or ACL injury at the senior level. Nevertheless, as the ACL injury burden indeed involves a higher risk of a second ACL injury [11, 32], the presented prevalence data highlight a tremendous concern regarding the senior level.

Third, although eight players (1% of the total cohort) participated in both the 2012 and 2019 surveys, this does not affect the findings, as each survey was analyzed independently as a cross‐sectional sample based on the players’ age group and playing level at each respective time point.

Furthermore, a limitation of the study is that knee pain was not reported per limb. Knee pain or discomfort was reported only as present or absent, irrespective of whether it occurred in one or both limbs.

Finally, the study has no information about the players who did not respond to the questionnaire, which means we cannot rule out the risk of selection bias. However, the high response rates (73%) as well as players representing 121 different teams do support high external validity of the results.

## 5. Conclusion

The prevalence of ACL injury history in active female football is high. Among senior players, approximately 1 in 8 non‐elite players and 1 in 6 elite players have suffered an ACL injury. The strong association between ACL injury history and activity‐related knee pain documents that an ACL injury has a huge impact on both young and adult women’s knees.

## Author Contributions

Mette K. Zebis: conceptualization, data curation, formal analysis, methodology, project administration, supervision, and writing–original draft; Mikkel B. Clausen: conceptualization, data collection, validation, and writing–review and editing; Connie Linnebjerg: conceptualization, data collection, methodology, and writing–review and editing; Mette Hansen: data curation, formal analysis, supervision, and writing–review and editing; Jesper Bencke: conceptualization, data collection, methodology, and writing–review and editing; Lars L. Andersen: conceptualization, data collection, methodology, and writing–review and editing; Per Hölmich: conceptualization, data curation, methodology, supervision, and writing–review and editing; Kristian Thorborg: conceptualization, data curation, formal analysis, methodology, project administration, supervision, and writing–review and editing.

Mette K. Zebis and Kristian Thorborg serve as the guarantor and accept full responsibility for the conduct of the study and the finished work, had access to the data, and controlled the decision to publish.

## Funding

No funding was received for this research.

## Disclosure

All authors read and approved the final version of the manuscript.

## Conflicts of Interest

The authors declare no conflicts of interest.

## Supporting Information

STROBE Checklist.

## Supporting information


**Supporting Information** Additional supporting information can be found online in the Supporting Information section.

## Data Availability

The data that support the findings of this study are available from the corresponding author upon reasonable request.
